# Recent Advances of Natural-Polymer-Based Hydrogels for Wound Antibacterial Therapeutics

**DOI:** 10.3390/polym15153305

**Published:** 2023-08-04

**Authors:** Yue Zhao, Xiaoyu Wang, Ruilian Qi, Huanxiang Yuan

**Affiliations:** 1 Department of Chemistry, College of Chemistry and Materials Engineering, Beijing Technology and Business University, Beijing 100048, China; 2 School of Materials Science and Engineering, University of Science and Technology Beijing, Beijing 100083, China

**Keywords:** hydrogel, natural polymer, antibacterial, wound healing

## Abstract

Hydrogels have a three-dimensional network structure and high-water content, are similar in structure to the extracellular matrix, and are often used as wound dressings. Natural polymers have excellent biocompatibility and biodegradability and are commonly utilized to prepare hydrogels. Natural-polymer-based hydrogels can have excellent antibacterial and bioactive properties by loading antibacterial agents or being combined with therapeutics such as phototherapy, which has great advantages in the field of treatment of microbial infections. In the published reviews of hydrogels used in the treatment of infectious wounds, the common classification criteria of hydrogels include function, source of antibacterial properties, type of antibacterial agent, etc. However, there are few reviews on the classification of hydrogels based on raw materials, and the description of natural-polymer-based hydrogels is not comprehensive and detailed. In this paper, based on the principle of material classification, the characteristics of seven types of natural polymers that can be used to prepare hydrogels are discussed, respectively, and the application of natural-polymer-based hydrogels in the treatment of infectious wounds is described in detail. Finally, the research status, limitations, and prospects of natural-polymer-based hydrogels are briefly discussed.

## 1. Introduction

The skin is the largest organ of the human body, which can protect the organism from external damage, but it is also prone to wounds [1,2]. According to the pathogenesis and effects of wounds, they can be divided into acute wounds and chronic wounds [3]. In particular, chronic wounds that are usually caused by endocrine diseases such as diabetes and neurological diseases have a profound impact on the quality of life as cardiovascular diseases and are particularly difficult to heal [4,5]. According to the Global Wound Care Market report released in 2016, the global wound care industry is expected to be worth 26.24 billion USD by 2023 [6]. Improper wound care is prone to microbial infection, causing long-term inflammation and making the wound difficult to heal [7]. More than 80 years ago, Alexander Fleming discovered penicillin, which made it possible to treat diseases caused by microbial infections [8]. However, the abuse of antibiotics has led to the gradual development of resistance in various pathogens [9], such as vancomycin-resistant *Enterococcus* (VRE) and multidrug-resistant *S. aureus* (MRSA) [10], making the wound more difficult to heal. Therefore, how to effectively treat chronic wounds caused by multidrug-resistant bacteria is a challenge.

**Figure 1:**
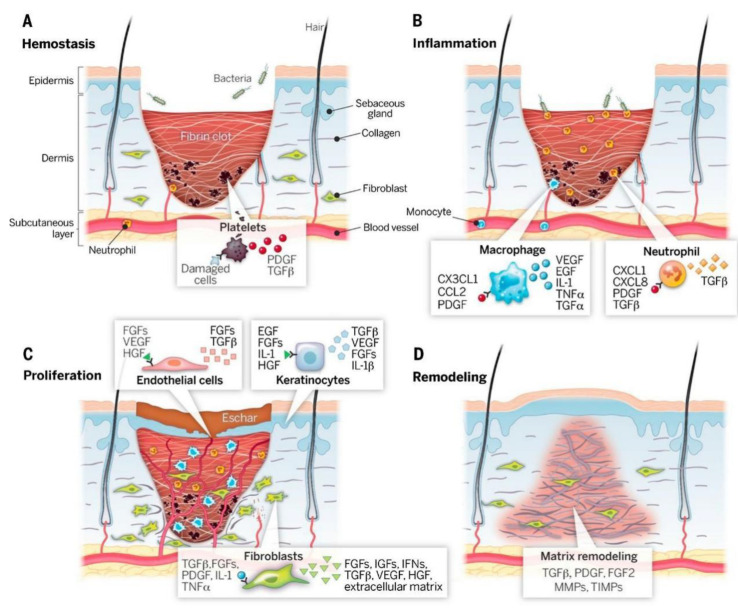
Four stages of wound healing: (**A**) hemostasis, (**B**) inflammation, (**C**) proliferation, (**D**) remodeling. Reprinted with permission from Ref. [11]. Copyright 2018 Elsevier Ltd.

As shown in Figure 1 [11], wound healing undergoes the following four processes: hemostasis, inflammation, proliferation, and remodeling, among which a variety of growth factors, enzymes, and cytokines play an important role in the synergistic regulation of cell activity [12]. Generally, acute wounds can heal normally, but chronic wounds are susceptible to various adverse factors such as microbial infection [13] and allergy [14], which makes the wound healing process more difficult to proceed smoothly. For example, during the repair process of a diabetic wound, due to long-term stimulation of hyperglycemia, ROS, and proteases (such as matrix metalloproteinases), the wound tissue will be affected by water loss, microbial infection, fibroblast migration, and reduced proliferation, and it will be difficult to heal [15,16].

In recent years, strategies based on bioactive materials such as electrospinning and hydrogels combined with phototherapy have been used to treat resistant bacterial infections and have achieved excellent therapeutic effects [17,18,19]. Particularly, hydrogels have a three-dimensional network structure and high-water content, similar in structure to extracellular matrix (ECM), which is a good wound dressing and can effectively heal wounds [20,21]. However, there are many defects in traditional hydrogels, such as poor mechanical properties, only a wet environment, and the inability to mimic natural tissue microstructure, which limit their application in wound treatment [19,22]. As a wound dressing, hydrogels should meet the following requirements [23,24,25]: (1) Good biocompatibility and non-cytotoxicity without causing wound allergy and inflammation; (2) Good moisture and moisture absorption which can maintain the wet environment of the wound to promote cell hydration and absorb the tissue exudate of the wound; (3) Good mechanical properties for perfectly attaching to the surface of the wound to form a barrier to prevent secondary infection by microorganisms; (4) Easy to peel without causing wound pain and secondary damage; (5) Good antibacterial, anti-inflammatory, antioxidant and hemostatic effects; (6) Induction of fibroblast adhesion and proliferation to promote wound tissue regeneration.

In recent years, the research on hydrogels has shown an increasing trend, and their functions have become more and more abundant [26,27,28]. For example, Liu et al. [29] and Fu et al. [30] achieved high antimicrobial efficiency by loading antibacterial agents into hydrogels or by combining them with phototherapy. Han et al. [31] used bionic mussels to achieve high viscosity and antibleeding to form a barrier to prevent microbial infection and achieve painless peel after adding strong oxidants without causing secondary damage to the skin. Shao et al. [32] effectively cleared ROS by introducing boron ester bonds to alleviate oxidative stress. Shen et al. [33] cross-linked hydrogels with dynamic acylhydrazone bonds to engender self-healing and luminescence properties of hydrogels, accompanied by loading growth factors to promote tissue regeneration. However, at present, hydrogels are difficult to achieve multifunctional integration and usually need to load various active substances, resulting in poor biocompatibility and low clinical application potential.

The antibacterial properties of hydrogels are related to the type of loaded antibacterial drugs, the antibacterial therapy, and the mode of drug release. Common loaded drugs include inorganic metals and antibiotics. Among all of the inorganic antibacterial agents, Ag is the most widely used because of its low biological toxicity and good anti-inflammatory and antibacterial properties [34]. Its antibacterial effect is affected by the size of the Ag-loaded hydrogel [35]. Antibiotics that are loaded into hydrogels can reduce drug dosage and improve bioavailability, but there is still a potential risk of drug resistance [10]. Hydrogels can also be used in synergy with photothermal therapy and photodynamic therapy to improve antibacterial activity [30,36]. Photothermal therapy refers to the antibacterial method of photothermal agents by generating thermal effects under the irradiation of near-infrared light [37]. Photodynamic therapy refers to the method of inducing bacterial death via producing ROS by photosensitizer under light of a specific wavelength [38]. The above two therapies combined with hydrogel have the advantages of being non-invasive, high selectivity, and negligible drug resistance, which has been widely used in antimicrobial infection [39,40,41]. Cross-linking methods of hydrogels, such as π-π stacking and hydrogen bonding, can realize the controlled release of drugs, thereby improving the antibacterial properties [42].

Synthetic polymers, such as polyethylene glycol, polyvinyl alcohol, and polyacrylamide, have good solubility and biocompatibility and are easy to modify, so they are often used in the preparation of hydrogels [43,44,45].

Natural polymers are macromolecular organic compounds that naturally exist in living organisms, such as polysaccharides and proteins [46]. Due to its excellent biocompatibility and biodegradability, it has been widely used in hydrogel wound dressings, with common materials including chitosan (CS), polydopamine (PDA), gelatin, agarose, sodium hyaluronate (HA), cellulose, and alginate [47,48,49]. Based on the classification principle of raw materials, the application of natural-polymer-based hydrogels in the treatment of infectious wounds was reviewed in this paper, which is summarized in Table 1. The study of natural macromolecular hydrogels is of great significance for the treatment of wounds, especially those infected by drug-resistant microorganisms. In addition, the research status, limitations, and prospects of natural-polymer-based hydrogels are briefly discussed.

## 2. Hydrogels Based on Natural Polymer Matrix for Antibacterial Therapy of Wound Infections

### 2.1. Chitosan (CS)

Chitosan (CS), a biological polysaccharide extracted from natural chitin, carries a positive charge at physiological pH and is usually present in the shell of arthropods, crustaceans, and fungal cell walls [64,65]. CS has good biocompatibility, biodegradability, bioactivity, and adhesion [66,67]. In addition, CS contains a positive charge and can play a certain killing effect on microorganisms through electrostatic interaction [68]. However, its low solubility and poor mechanical properties limit its clinical application [69]. Hydrogels with good performance can be prepared by modifying the active hydroxyl and amino groups on CS, which can be used for efficient wound treatment [70,71].

**Figure 2:**
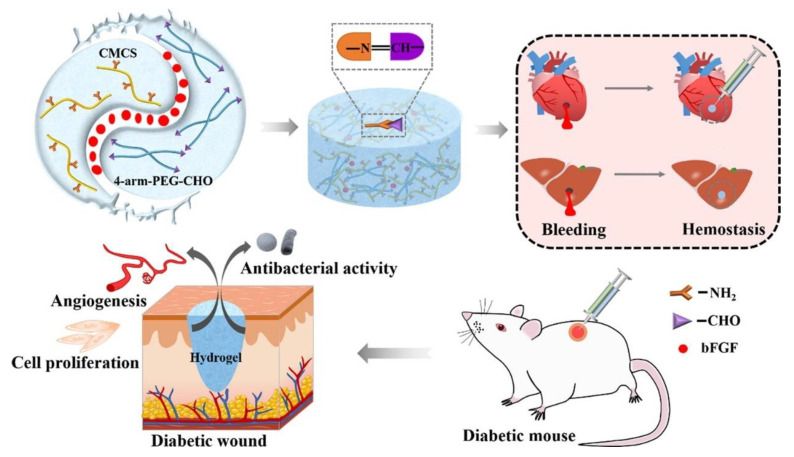
The illustrated preparation process of BP/CS-bFGF hydrogel and the application in promoting diabetic wound healing in mice. Reprinted with permission from Ref. [50]. Copyright 2022 Elsevier Ltd.

Hao et al. [50] designed an injectable BP/CS-bFGF hydrogel to mediate associated cellular responses and promote full-layer diabetic wound healing (Figure 2). In this study, CS was modified to obtain carboxymethyl chitosan (CMCS) to enhance its water solubility and biocompatibility. Compared with straight-chain polyethylene glycol (PEG), branched-chain PEG has stronger mechanical properties and hemostatic properties. Therefore, 4-arm-PEG-CHO and CMCS were selected to form a covalently cross-linked network through Schiff base reaction, and BP/CS hydrogel was obtained. At the same time, the basic fibroblast growth factor (bFGF) is loaded into it to promote fibroblast proliferation, extracellular matrix fibrosis, and blood vessel formation. The swelling rate of BP/CS-bFGF hydrogel is as high as 132% at 37 °C, which can absorb a large amount of tissue exudate and maintain the humidity of the wound environment. The hydrogel has good self-healing ability, and after injection into irregular wounds located in joints, it can be rapidly shaped into defective shapes to enhance the healing of target tissues. Hydrogels have a good hemostatic effect due to their high adhesion and the presence of CMCS. Antibacterial experiments showed that the antibacterial rate of hydrogel for *E. coli* and *S. aureus* was more than 67%. This is because CMCS has a positive charge and can be adsorbed to the surface of bacteria through electrostatic interactions, inhibiting the replication of bacterial DNA. The hydrogel also has good blood compatibility and cell compatibility. Hydrogel was injected into the diabetic wounds of mice. The hydrogel quickly adhered to the wound and stopped bleeding, creating a good healing environment for the wound, while bFGF was released to promote tissue repair. After 14 days of treatment, the expressions of CD31, CD34, and Ki67 increased significantly, and the wound healing rate was 99%. In conclusion, BP/CS-bFGF hydrogel mimics the pathological environment of the extracellular matrix to effectively heal wounds by releasing the bioactive molecule bFGF and antibacterial properties. This work was the first to investigate the effect of hydrogels loaded with basic fibroblast growth factor (bFGF) on diabetic full-layer wound repair. It provides a reference for the treatment of diabetic chronic wounds. However, the antibacterial properties of the hydrogel come from the electrostatic force between CMCS and bacteria, which is far from enough to deal with serious infections, and it is necessary to load antibacterial agents or combine them with other therapies to improve the antibacterial activity.

Although hydrogels can promote wound repair by inducing tissue regeneration, there is still a risk of microbial infection if the desired antibacterial effect is not achieved, which can affect wound healing. Therefore, it is very important to develop a kind of hydrogel with an excellent antibacterial effect. Yang et al. [51] developed a highly antibacterial and antioxidant CSCG-PEG/DMA/Zn hydrogel by adding the antibacterial agent Zn^2+^, which simultaneously promoted the regeneration of blood vessels and hair follicles and further promoted wound healing. CS was modified with PEG to obtain water-soluble and biocompatible CS-PEG, then the double bond was introduced by grafting glyceryl methacrylate to make it have the ability of photoinitiated polymerization, which was cross-linked with dopamine (DMA) through photoinduced free radical polymerization to form a double-network cross-linked hydrogel. At the same time, the antibacterial agent Zn^2+^ was loaded to improve the antibacterial properties. The double network structure makes the hydrogel have good mechanical properties, such as compressibility and resilience and can withstand large external forces. The degradation rate and swelling rate of the hydrogel can be adjusted by changing the content of DMA, which can absorb tissue exudate on demand while maintaining the humidity of the wound environment. The hydrogel has excellent antioxidant properties, and the DPPH clearance rate can reach more than 95%, which can effectively relieve the oxidative stress of wounds. Most importantly, after the co-culture of CSG-PEG/DMA/Zn hydrogels with bacteria at 37 °C for 2 h, the antibacterial rates of hydrogels containing 0.6% and 0.9% of DMA against *S. aureus*, MRSA, and *E. coli* reached 100%. Moreover, the slow release of Zn^2+^ makes the hydrogel possess long-term antibacterial properties. CSC-PEG/DMA6/Zn hydrogel was used to treat the wounds of MRSA-infected mice. On the 14th day, the wound closure rate was more than 95%, which was much higher than that of other control groups. CSG-PEG/DMA/Zn hydrogel has great potential in the treatment of infected wounds due to its excellent antimicrobial properties, biocompatibility, antibleeding and tissue adhesion. In this study, multifunctional hydrogels were prepared by photoinitiation polymerization, which provided a new idea for wound dressing research.

### 2.2. Polydopamine

Polydopamine (PDA) is an important natural melanin analog produced by dopamine autoxidation, which is easy to prepare and has good photostability, biodegradability, and biocompatibility [72,73,74]. The dynamic bonds based on catechol groups in PDA are self-healing and can be easily broken, making the hydrogel tough and stretchable [75]. PDA can mimic mussels to produce highly viscous hydrogels. Based on this property, Di et al. [76] designed a highly viscous, reusable, and heat-reversible hydrogel for the preparation of wound dressings, electronic skin, and wearable devices. In addition, PDA also has excellent photothermal properties, which can destroy the structure and function of cancer cells and microorganisms through photothermal therapy (PTT) to effectively treat cancer and microbial infection [77,78]. Therefore, PDA-based hydrogels can be loaded with antibacterial agents or combined with PTT to achieve high antibacterial efficiency. However, when PDA is used as a photothermal agent (PTA), there are two defects that limit its application. First, the photothermal conversion efficiency (PCE) is relatively low (about 20%) under the irradiation of an 808 nm near-infrared (NIR) laser, and PCE decreases with the extension of wavelength. Second, there are a large number of active groups on the surface of PDA, such as catechol, primary amine, and secondary amine groups, which make it easy to aggregate, reduce light absorption efficiency, and thus reduce PCE [79]. Therefore, when PDA is used as PTA, how to improve the PCE of PDA and prevent its aggregation is the key to achieving excellent antibacterial properties of photothermal hydrogels.

Liu et al. [52] developed a multifunctional hydrogel CAC/PDA/Cu (H_2_O_2_) consisting of CMC, alginate, and PDA with antibacterial, anti-inflammatory, and excellent mechanical properties by loading bionic mussel. PDA has strong metal ion trapping properties so that Cu^2+^ can be quickly and evenly distributed on the porous hydrogel surface. The hydrogel has excellent mechanical capacity, and its elongation at break (77.93 ± 4.02%) is greater than that of natural skin (70 ± 5%), which can ensure integrity and mechanical strength when applied to the skin surface. The interconnected high-porosity structure of hydrogels can absorb tissue exudate, maintain wound humidity, and ensure the transport of nutrients and the exchange of biomolecules within cells. PDA/Cu (H_2_O_2_) coating can induce fibroblasts to migrate to the vicinity of the wound, promote angiogenesis, and thus accelerate tissue repair. The hydrogel can also inhibit inflammation, clear ROS, and improve the wound microenvironment. In vitro antibacterial experiments showed that hydrogels had strong antibacterial activities against *S. aureus* (99.87%), *E. coli* (99.14%), and MRSA (99.25%). In addition, the bactericidal rate of hydrogel against MRSA in vivo was almost 100%, and the wound healing rate was 97.86% on the 14th day. This is mainly because both CMC and Cu^2+^ have antibacterial properties and can enhance the antibacterial effect through synergistic action. In summary, the hydrogel has high anti-inflammatory, antibacterial, and tissue repair-inducing abilities, and its excellent biocompatibility gives it clinical potential for treating resistant bacteria-infected wounds.

**Figure 3:**
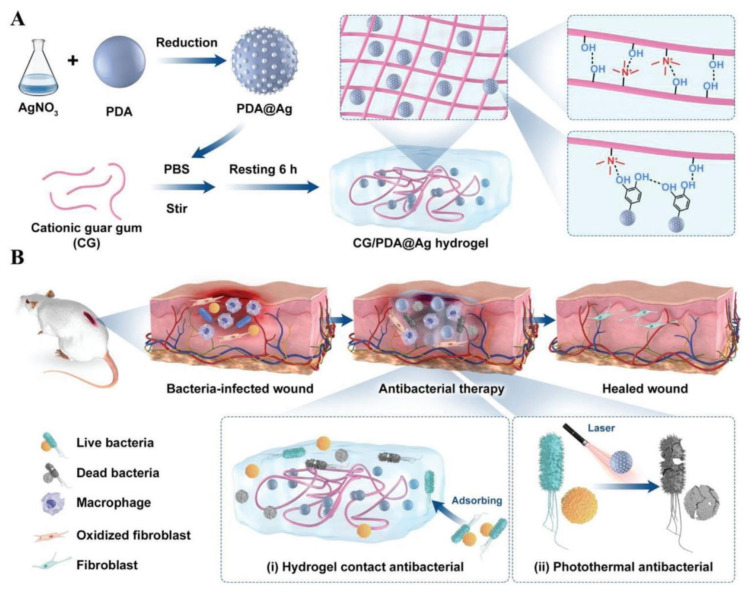
Schematic diagram of (**A**) the hydrogel synthesis mechanism and (**B**) photothermal/chemo-dynamic synergistic treatment of MRSA-infected wound. Reprinted with permission from Ref. [53]. Copyright 2022 the authors.

Although PDA-based hydrogels containing antibacterial agents can achieve antibacterial purposes, the preparation process is complicated and usually requires a variety of substances to occur cross-linking reactions. The method combined with PTT only requires a simple modification to achieve an excellent antibacterial effect and can prevent temperature during PTT from being too high to cause damage to normal tissue. Qi et al. [53] designed an efficient photothermal treatment nano-platform (CG/PDA@Ag hydrogel) for efficient photothermal antibacterial therapy and promoting wound healing (Figure 3). The charge transfer efficiency and non-radiative transition of PDA@Ag nanoparticles are improved by using Ag for the modification of PDA, and the PCE is increased from 16.6% (PDA) to 36.1%. PDA@Ag nanoparticles were uniformly distributed in CG/PDA@Ag hydrogel without aggregation, and PCE was further increased to 38.2%. The existence of non-covalent bonds in hydrogels, such as hydrogen bonds, π-π stacking, and electrostatic force, make them have good mechanical properties and self-repair capability, which is conducive to injection and prolongation of use time. Cationic guar gum (CG) contains a large number of hydroxyl and quaternary ammonium groups, which are positively charged and can trap negatively charged bacteria through electrostatic interaction and hydrophobic interaction to play a bacteria-killing role. Ag^+^ can be released continuously for up to 6 days, giving the hydrogel a long-term bactericidal effect. After 3 min of NIR irradiation (808 nm, 1 W·cm^−2^), the temperature of CG/PDA@Ag hydrogel could be elevated to 67.3 °C, and the inhibition rates against *E. coli* and *S. aureus* were 99.9% and 99.8%, respectively. Under the combined treatment of CG, Ag^+^, and light irradiation, the cell membranes of these two strains of bacteria were severely damaged. Consistent with the results of in vitro experiments, the hydrogel can also play an effective antibacterial role in vivo with good biocompatibility, which can promote rapid wound healing. In addition, organisms can absorb CG/PDA@Ag hydrogels without additional removal. In short, CG/PDA@Ag hydrogel overcomes the shortcomings of PDA and has efficient PCE and strong bacteria trapping/killing ability to effectively promote wound healing in a short time. This research has made a breakthrough in the modification of PDA photothermal agents and realized practical application.

### 2.3. Gelatin

Gelatin is a natural polymer with a collagen sequence, in which the amino acid sequence RGD (Arg/Gly/Asp) can promote cell adhesion and is conducive to cell growth [80]. Due to its good biocompatibility, biodegradability, fibroblast adhesion, and proliferation characteristics, it has great potential in biomedical and tissue engineering applications [81,82]. However, the poor mechanical property, easy fracture, and rapid degradation of gelatin in the dry state limit its application as a hydrogel dressing [83,84]. Recent reports found that hydrogels prepared by using polysaccharide derivatives containing aldehyde group as cross-linking agents and Schiff base reaction between an aldehyde group and amino group of gelatins have both good mechanical properties and little biological toxicity [85,86].

**Figure 4:**
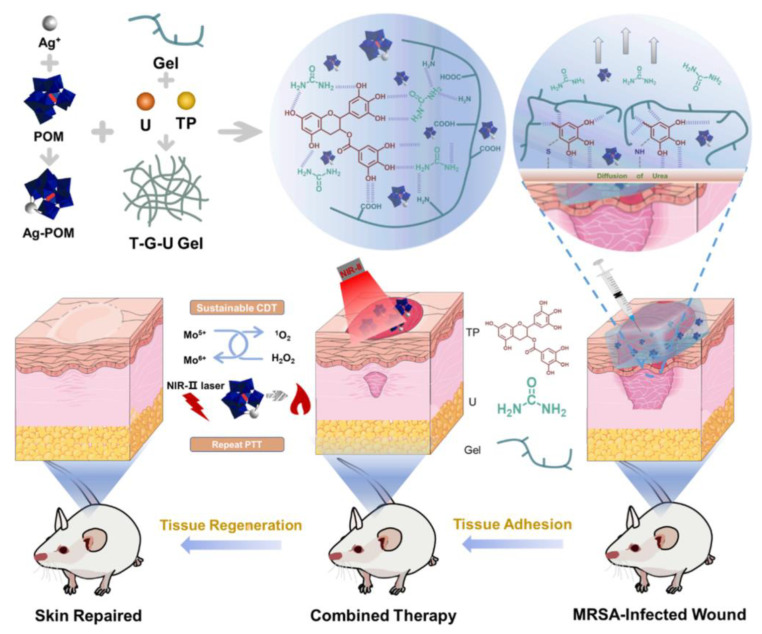
Illustration of the hydrogel synthesis mechanism and photothermal/chemodynamic synergistic treatment of MRSA-infected wound. Reprinted with permission from Ref. [54]. Copyright 2023 American Chemical Society.

Huang et al. [54] combined hydrogels with PTT and chemodynamic therapy (CDT) to design a bacterial environment-responsive multifunctional injectable hydrogel for treating bacterial infections and promoting wound healing (Figure 4). The natural component tea polyphenols (TPs) were used as cross-linking agents to form a highly cross-linked network with gelatin polymer. At the same time, urea was added to make the hydrogel injectable and have good mechanical properties. Silver polyoxometalate (AgPOM) that is doped in the hydrogel is protonated and aggregated in an acidic environment to exhibit good photothermal performance under NIR-II irradiation. In addition, Mo^5+^ in AgPOM reacts with overexpressed H_2_O_2_ in the wound to produce ^1^O_2_, which achieves a high antibacterial effect in synergy with PTT. The hydrogel containing AgPOM can react with additional H_2_O_2_ to produce ^1^O_2_, and combined with the photothermal effect of AgPOM under NIR-II light irradiation for 10 min (1060 nm, 1 W·cm^−2^), the antibacterial rate of the hydrogel against MRSA both in vivo and in vitro can reach more than 90%. Importantly, the good adhesion of the hydrogel also makes it have long-term antibacterial activity. Due to the excellent biocompatibility and antibacterial properties of the hydrogel, a large amount of collagen was deposited on the wound of the mice after 14 days of treatment, and the wound healed almost completely. In conclusion, the hydrogels prepared in this study combined PTT and CDT and opened up new thinking for the development of multifunctional hydrogels.

When microorganisms infect wounds, they tend to integrate communities and embed themselves in extracellular polymeric substances (EPS), forming a dense structure called biofilm [87]. As a barrier, EPS can resist physical and chemical damage, so ordinary methods such as antibiotics cannot effectively eliminate biofilm, resulting in long-term microbial infection and difficult wound healing [88,89]. Eliminating biofilms is, therefore, extremely important for treating wounds. Wang et al. [55] designed a photothermal bilayer GelMA-EGF/Gelatin-MPDA-LZM hydrogel dressing for efficient biofilm elimination and comprehensive treatment of chronic wounds. The outer layer of hydrogel was photo cross-linked to obtain GelMA with good mechanical properties for the adhesion and proliferation of fibroblasts. Meanwhile, EGF was added to GelMA to promote tissue regeneration and wound re-epithelialization. The mesoporous polydopamine (MPDA) nanoparticles with high PCE were loaded with lysozyme (LZM) to form the inner hydrogel. After 10 min of NIR light irradiation (808 nm, 1 W·cm^−2^), the photothermal effect of MPDA can destroy and eliminate the biofilm structure, making LZM penetrate into the biofilm and kill bacteria with an antibacterial rate in vivo as high as 98.08%, and the wound temperature only increased to 46 °C which cannot induce damage to the tissue around the wound. SEM observation showed that after photothermal treatment, there was almost no biofilm structure in the wound tissue, and the bacterial morphology was destroyed. After 12 days of treatment, the wound infected with *E. coli* remained at only 7.9% of the area. This bilayer hydrogel can effectively eliminate the biofilm and kill bacteria under the cooperation of photothermal therapy and has a broad application prospect in promoting the repair of infectious chronic wounds.

### 2.4. Agarose

Agarose is a naturally water-soluble linear polysaccharide found in seaweed that can be dissolved in neutral or alkaline solutions [90]. It has excellent biocompatibility, gelling properties, and physicochemical characteristics, so it is often used as a biomaterial for cell growth and local drug delivery [91,92], but it is not widely used in antibacterial hydrogels. The agarose can form a stable thermo-reversible gel by physical cross-linking without additional cross-linking agents, and the gel temperature is about 25 °C, which is suitable for the preparation of wound dressing [93,94]. However, the low chemical complexity of agarose limits its direct application in fine chemical and biological reactions, and it can be modified by biological, physical, and chemical methods [91,95].

**Figure 5:**
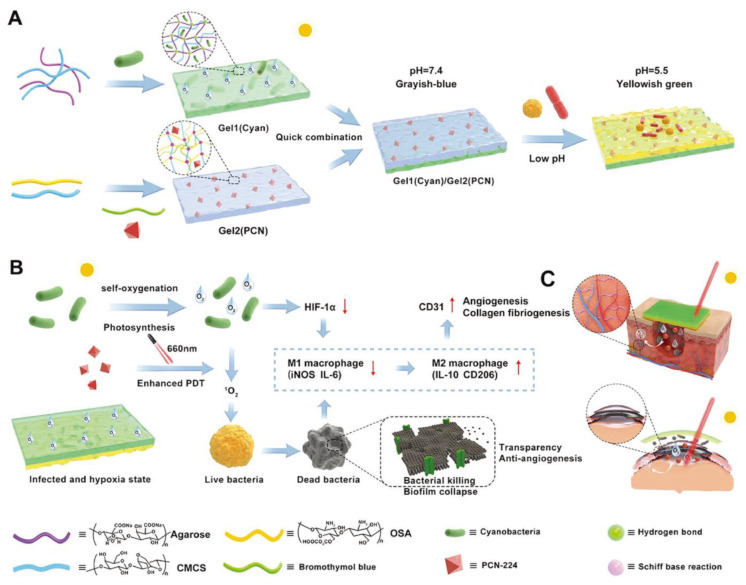
(**A**) Illustration of (**A**) the synthesis of Gel1 (Cyan)/Gel2 (PCN) hydrogel, (**B**) properties of Gel1 (Cyan)/Gel2 (PCN) hydrogel and (**C**) the application of Gel1 (Cyan)/Gel2 (PCN) hydrogel in diabetic wound and keratitis healing. Reprinted with permission from Ref. [56]. Copyright 2022 Wiley-VCH GmbH.

Damaged blood vessels and high metabolic demand can lead to a severe shortage of oxygen supply to diabetic chronic wounds, resulting in more difficult wound healing [96]. Among the existing methods, hyperbaric oxygen therapy and local oxygen therapy have little effect. Although local dissolved oxygen therapy can improve oxygen penetration and absorption, it is dependent on hyperbaric oxygen generators and has a high risk of wound infection [97]. In addition, the high-sugar microenvironment is also conducive to microbial growth, resulting in chronic inflammation and necrosis of capillaries and nerve endings [56]. Therefore, the provision of oxygen to the wound, as well as real-time monitoring and timely sterilization, is essential. Zhu et al. [56] designed a multifunctional double-layer hydrogel that could monitor wound conditions and continuously generate oxygen to enhance the antibacterial effect of PDT (Figure 5). First, sodium alginate/carboxymethyl chitosan is combined with photosensitizer (PCN-224) and pH indicator (bromothymol blue BTB) through Schiff base reaction to obtain the inner hydrogel Gel2. Under white light, PPCN-224 produces ROS, which destroys bacterial cell walls and effectively kills bacteria. Then, the hydrogel obtained by cross-linking agarose and CMCS is loaded with cyanobacteria to obtain the outer hydrogel Gel1. When an infection occurs in the wound, the pH drops, and the color of bromothymol blue (BTB) in the inner Gel2 gradually changes from gray-blue to yellowish-green, indicating the occurrence of a bacterial infection. At the same time, in an acidic environment, the Schiff base bond breaks, and PCN-224 is released, avoiding the loss of bactericide due to premature release. Under natural light conditions, the cyanobacteria in the outer Gel1 can continuously produce oxygen to relieve tissue hypoxia and enhance the antibacterial efficiency of PDT. Adequate oxygen in the environment also has other advantages, such as accelerating cell migration, alleviating inflammation, promoting skin capillary formation, and wound tissue recovery. The self-oxygenated double-layer hydrogel can synergistically treat the refractory wounds infected by anaerobic bacteria and repair the tissue in time through infection monitoring. Chronically infected wounds in mice were largely completely healed after 21 days of treatment with Gel1 (Cyan)/Gel2 (PCN) hydrogel under conditions of white light irradiation. However, it can be seen from the staining results of live/dead bacteria that a certain number of bacteria still survive after 5 min of illumination at 606 nm with the fluence of 200 mW·cm^−2^. Thus, the antibacterial effect of this method still needs to be strengthened. This study solves the problem of hypoxic infection of chronic wounds and provides insight into real-time monitoring and intelligent treatment of wounds.

**Figure 6:**
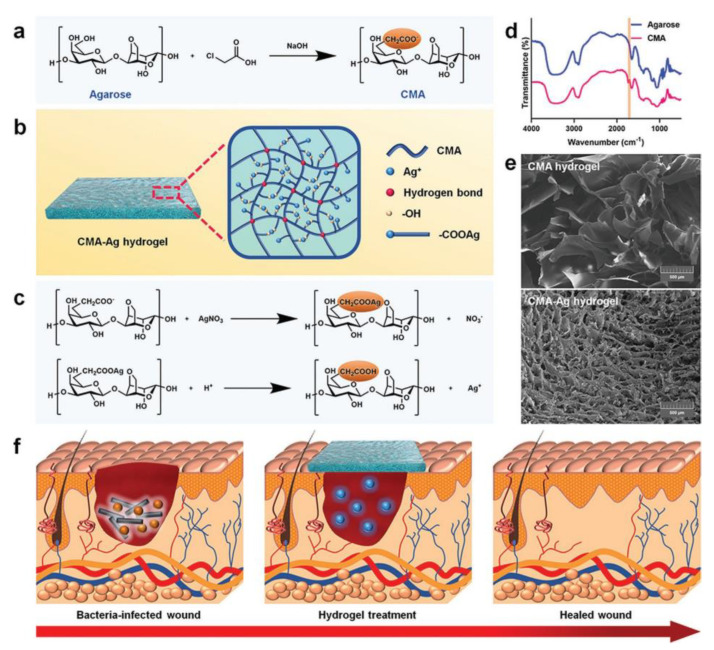
Illustrated preparation of (**a**) CMA molecule and (**b**) CMA–Ag hydrogel. (**c**) The mechanism of Ag^+^ releasing from CMA–Ag hydrogel. (**d**) FT–IR spectra of the agarose and CMA molecule. (**e**) Morphological characterization of CMA and CMA–Ag hydrogels. (**f**) Schematic application of CMA–Ag hydrogel in the treatment of infected wounds. Reprinted with permission from Ref. [57]. Copyright 2020 WILEY-VCH Verlag GmbH & Co. KGaA, Weinheim.

Huang et al. [57] designed a macroporous CMA-Ag hydrogel for accelerating wound healing with antibacterial and anti-inflammatory functions (Figure 6). Agarose was modified by introducing carboxymethyl to obtain carboxymethyl agarose (CMA), which improved the surface property of hydrogel and promoted cell proliferation. The carboxymethyl group on CMA deprotonates and interacts with Ag^+^, and the hydroxyl group also coordinates with Ag^+^ to obtain CMA-Ag hydrogel. The network formed by CMA and Ag^+^ coordination makes the porous structure of hydrogel denser with a larger swelling rate, and the tissue exudate can be absorbed more quickly. Low pH will destroy the ionic interaction between hydrogels, and higher temperatures will break the coordination. Therefore, the Ag^+^ release of the hydrogel is both pH- and temperature-responsive, and its release decreases with the increase in pH during wound healing and increases with the elevation of temperature. A continuous release can be achieved under body temperature conditions. The antibacterial performance of CMA-Ag hydrogel on *E. coli* and *S. aureus* increased with the increase in Ag^+^ content, and the antibacterial rate could reach as high as 100%. At the same time, in vivo experiments, the hydrogel also has a strong antibacterial activity (100%) against *S. aureus*. The *S. aureus* infected wound healed completely after 14 days of treatment with CMA-Ag hydrogel. CMA-Ag hydrogel can accelerate wound healing by relieving inflammation and promoting tissue regeneration and collagen deposition in the wound. The hydrogel prepared in this study is pH- and temperature-responsive, which can promote the early occurrence and end of inflammation and provide a certain reference for the rapid healing of infected wounds.

### 2.5. Hyaluronic Acid

Hyaluronic acid (HA) is a non-sulfonated glycosaminoglycan and natural anionic polysaccharide consisting of repeated disaccharide units of β-D-glucuronic acid and *N*-acetyl-D-glucosamine linked by alternating β-1, 3, and β-1, 4-glucosidic bonds, which are produced during fibroblasts proliferation [98,99,100]. HA can promote wound healing by promoting cell migration and mediating cell signal transduction and has good biocompatibility, biodegradability, and moisture retention [100]. HA can promote the formation of blood clots, the expression of interleukin, the migration and proliferation of keratinocytes and fibroblasts [101], and accelerate the formation and re-epithelialization of granulation tissue. It can also reduce inflammatory cell infiltration and promote wound healing [102].

**Figure 7:**
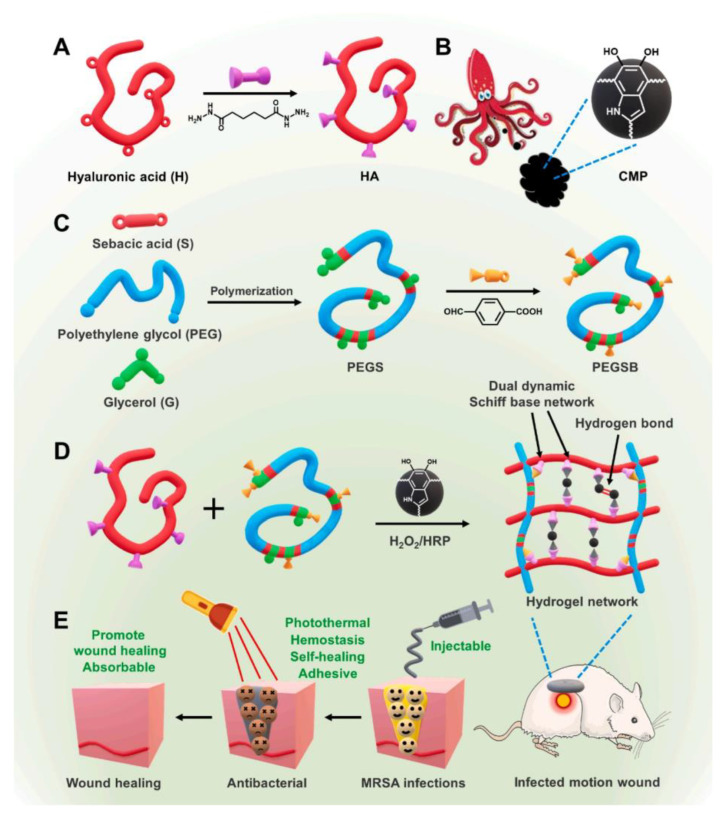
Schematic diagram of the synthesis of HA-PEGSB-CMP hydrogel and its application in promoting wound healing. (**A**) The synthesis of HA. (**B**) The structure of CMP. (**C**) The synthesis of PEGSB. (**D**) The preparation of HA-PEGSB-CMP hydrogel. (**E**) The wound healing application of the hydrogels. Reprinted with permission from Ref. [58]. Copyright 2021 Elsevier B.V.

The skin at the joint is frequently stretched, and traditional non-self-healing wound dressings are easy to damage or fall off, so the wound at this site is not easy to heal and is also susceptible to microbial infection [103]. Therefore, it is extremely important to design a wound dressing that not only has excellent mechanical properties but also can effectively realize antibacterial purpose and promote wound healing. Li et al. [58] designed an HA-PEGSB-CMP hydrogel with adhesion, self-healing, and antibacterial capabilities for the treatment of sports wounds (Figure 7). In this study, HA, as the main component, cross-linked with benzaldehyde functionalized PEG co-polyglycerol caprate (PEGSB) by Schiff base reaction to obtain HA-PEGSB hydrogel that has stretch ability and self-healing properties. The aldehyde group on PEGSB can react with the amino group of wound tissue to introduce tissue adhesion. HA-PEGSB was cross-linked with cuttlefish melanin nanoparticles (CMP) with mussel-like protein structure to obtain HA-PEGSB-CMP hydrogel with enhanced viscosity. A good stretching ability and self-healing capability make the hydrogel adhere firmly to different shapes of wounds. The excellent photothermal performance of CMP endows the hydrogel with antibacterial activity. After 3 min of NIR irradiation (808 nm, 0.8 W·cm^−2^), *E. coli* and MRSA are killed. The amino group in the dynamic Schiff base and the catechol structure of CMP can promote blood coagulation and give hydrogels good hemostatic properties, which can stop wound bleeding at 88 s. After 14 days of treatment on the hip joint wound of mice, the wound healed almost completely, and the skin surface was smooth. The good biocompatibility of the hydrogel makes it have great potential for clinical application to treat infected sports wounds.

**Figure 8:**
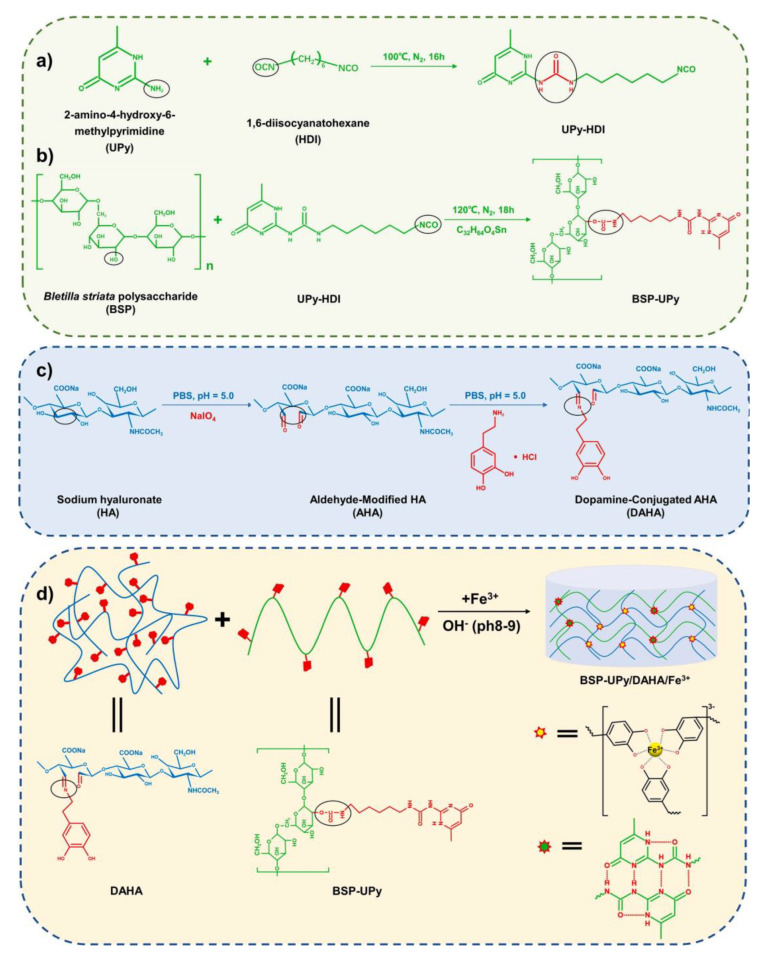
Schematic preparation of (**a**) UPy-HDI, (**b**) BSP-UPy, (**c**) DAHA and (**d**) BSP-U/DAHA hydrogels. Reprinted with permission from Ref. [59]. Copyright 2023 Elsevier Ltd.

Yue et al. [59] designed a dual-network multifunctional hydrogel based on polysaccharide materials for the treatment of wounds infected with drug-resistant bacteria (Figure 8). The first hydrogel structure of BSP-U with better physical and chemical properties was obtained by grafting UPy onto the main chain of Bletilla striata polysaccharide (BSP) with the premise of preserving the structure of supramolecular uracymidone (UPy) tetrahydric array. The second hydrogel structure was obtained by grafting dopamine hydrochloride (DP) to aldehyaluronate sodium (AHA) by Schiff base reaction. The quadruple hydrogen bonds of catechols, Fe^3+^, and UPy dimer form a highly dynamic double cross-linked network structure, which makes the hydrogel have good mechanical properties and can adhere to wound tissue well. The cross-linked structure is sensitive to light, heat, and acid and can achieve sol-gel transition and be easily removed. Hydrogel has excellent swelling, water retention, and air permeability, which can absorb tissue exudate and maintain the humidity and oxygen supply of the environment to create a good microenvironment for the wound. The hydrogels formed by the cross-linking of catechol compound and Fe^3+^ ligand can widely absorb NIR light and have photothermal properties. After 3 min of in vitro irradiation, the antibacterial rate of the hydrogels on *E. coli* and *S. aureus* can reach 91.68% and 94.94%, respectively, showing great potential in protecting wounds from microbial infection. In the wound treatment of *S. aureus*-infected mice wound, the antibacterial efficiency of hydrogel with the aid of NIR light reached 92.4%, and the wound healed almost completely on the 15th day. BSP-U/DAHA Hydrogel has excellent antibacterial ability and bioactivity, which is an ideal candidate for wound healing dressings of drug-resistant bacterial infections.

### 2.6. Cellulose

Cellulose is a kind of linear polysaccharide formed from D-glucose via a glycosidic linkage, mainly derived from plant cell walls and bacteria [104,105]. Cellulose has the advantages of good biocompatibility, biodegradability and regeneration, and good mechanical strength [106]. However, the poor solubility of cellulose limits its application [105]. Since cellulose is formed from glucose subunits, it is naturally biocompatible with human tissues and can be modified without affecting structural and mechanical properties [107]. Bacterial cellulose (BC), derived from Gram-negative bacteria, has strong hydrophilicity and a large specific surface area for great application potential [107,108].

**Figure 9:**
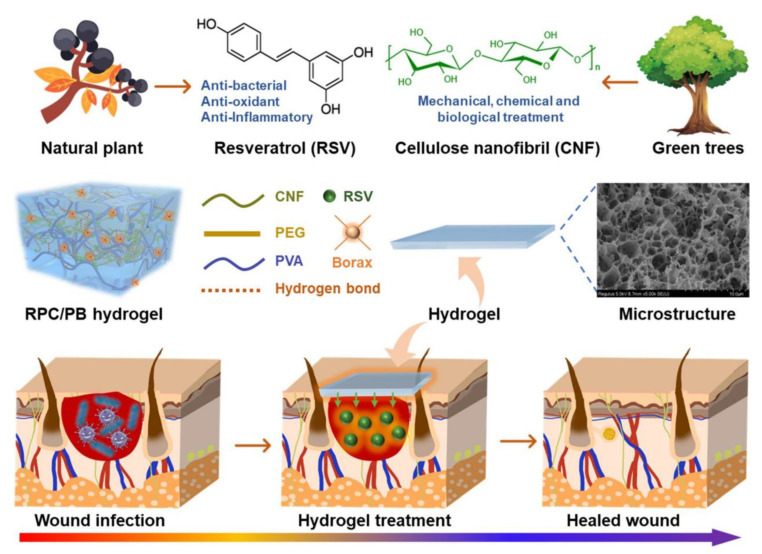
Schematic preparation and application of RPC/PB hydrogel in the treatment of infected wound. Reprinted with permission from Ref. [60]. Copyright 2022 The Authors.

The proliferation of microorganisms will lead to the acidity of the wound microenvironment, which can be used to prepare pH-responsive hydrogels that intelligently control drug release [109]. Yang et al. [60] designed a pH-responsive hydrogel for the treatment of infected wounds (Figure 9). First, the natural antibiotic resveratrol (RSV) was esterified by reacting with PEG to obtain RSV-PEG, and then the RPC-conjugated polymer was synthesized by amide reaction with cellulose nanofibers. RPC-conjugated polymers form semi-interpenetrating polymer networks and hydrogen bonds in PVA/Borax networks to enhance the mechanical strength and physicochemical properties of hydrogels. The degradation of RPC/PB hydrogels is pH-dependent, and RSV release can reach 64.2% when pH is 5.4. The dense, porous, and interwoven structure of hydrogel can absorb tissue exudate and increase oxygen permeability to promote wound healing. The physical interactions in the hydrogel network are reversible, allowing it to be reused and stripped without damaging the skin and to have a self-healing capacity. The antibacterial experiment showed that with the increase in RPC content, the antibacterial rate of hydrogel on *S. aureus* was up to 84.3%. The excellent antioxidant and antibacterial properties of RPC, as well as the good mechanical properties and biocompatibility of the hydrogel, allow the wound to heal almost completely after 12 days. This study achieved intelligent controlled drug release, which is expected to replace traditional hydrogels and achieve more extensive clinical application in infectious wounds.

**Figure 10:**
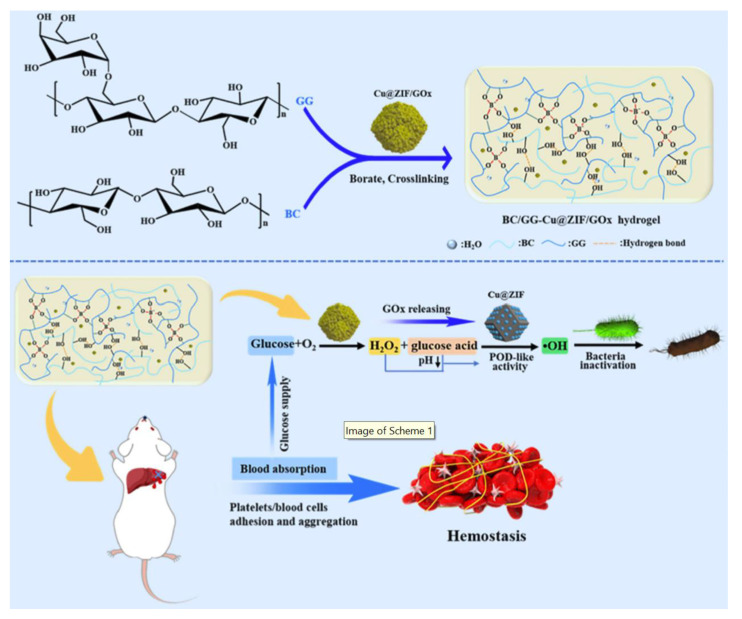
Illustrated preparation of BC/GG-Cu@ZIF/GOx hydrogel, and its application in antibacterial and hemostasis. Reprinted with permission from Ref. [61]. Copyright 2022 Elsevier Ltd.

ROS includes H_2_O_2_, •OH, ^1^O_2_, etc. Among them, highly active •OH can oxidize cell membranes and intracellular biomolecules (such as proteins and lipids) and has a stronger killing ability against bacteria than H_2_O_2_ to avoid microbial resistance [110]. Nano-enzymes can simulate the activity of peroxidase and convert H_2_O_2_ to •OH for the treatment of diseases caused by microbial infections [111]. Zhang et al. [61] designed a biomimetic hydrogel with the catalytic ability of glucose reaction for synergistic antibacterial and hemostatic use (Figure 10). The prepared Cu@ZIF/GOx was wrapped in a cross-linked network composed of bacterial cellulose (BC) and guar gum (GG) to form a mixed hydrogel. The hydrogel supported by glucose oxidase (GOx) can catalyze glucose at the wound site to form H_2_O_2_ and gluconic acid. The accumulation of gluconic acid will decrease the pH at the wound site and further activate the activity of Cu@ZIF nano-enzyme, which simulates POD to catalyze the decomposition of H_2_O_2_ to generate high cytotoxic •OH, which inactivates bacteria. Under the condition of glucose concentration of 10 mM, the inhibition rate of hydrogel on *E. coli* and *S. aureus* can reach 100%, and it can also effectively eliminate biofilms. The recombination of hydrogen bonds at the interface and the reversible recombination of borate diol make the hydrogel have good self-healing and flexibility. The superior water absorption and hydrophilicity of hydrogel can attract red blood cells and platelets to gather in the porous structure and quickly stop bleeding. The hydrogel prepared by this method is glucose-responsive and innovatively realizes the synergistic antibacterial hemostatic treatment of infected wounds through the glucose-catalyzed cascade reaction, providing a good reference for the treatment of diabetic wounds.

### 2.7. Alginate

Alginate is a natural anionic polysaccharide derived from various brown algae and bacteria [112,113]. It is widely used due to its advantages, such as excellent biocompatibility, nonimmunogenic, non-thrombogenic, and high safety, which is a kind of polysaccharide approved by the US Food and Drug Administration [114]. However, due to its high hydrophilicity, it is not conducive to drug-loading and releasing [62]. The hydroxyl and carboxyl groups on its main chain can be modified to make it amphiphilic, which can be used to stabilize the emulsion and load drugs [115,116].

**Figure 11:**
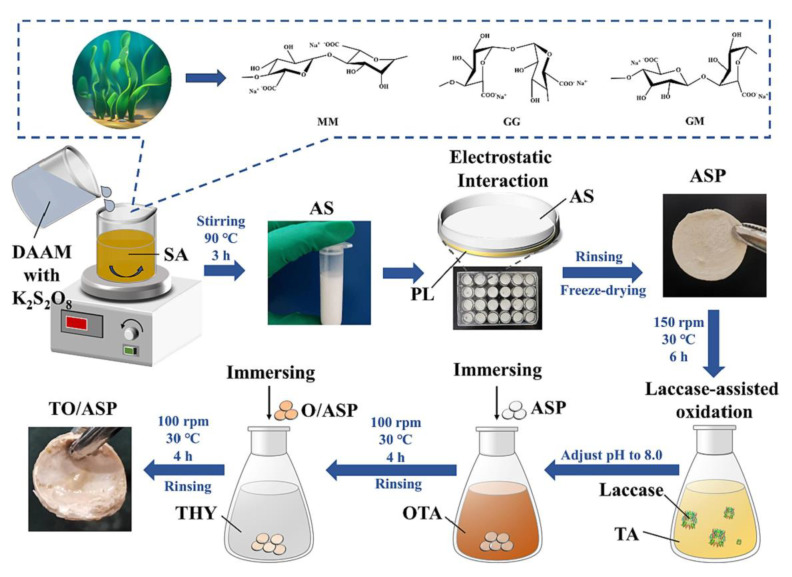
Schematic representation of TO/ASP hydrogel. Reprinted with permission from Ref. [62]. Copyright 2022 Elsevier Ltd.

After the wound is infected by microorganisms, the microenvironment becomes acidic due to the bacterial metabolism to produce lactic acid and acetic acid [117]. During the healing process, the pH will gradually increase and fluctuate in the weakly alkaline range. The antibacterial activity of the hydrogel material may be affected by pH, so it is necessary to prepare a wound dressing that is not influenced by pH. Jin et al. [62] designed TO/ASP hydrogel, a highly effective antibacterial platform without influence by pH. Sodium alginate was modified by diacetone acrylamide to obtain amphiphilic sodium alginate (AS), and then formed hydrogel film (ASP) with polylysine (PL) in solution. A TO/ASP film was formed by loading lipophilic thymol (THY), which is a natural antibacterial substance, and hydrophilic oligo-tannic acid (OTA) oxidized by laccase on the ASP (Figure 11). OTA has a polyphenol structure, which is easy to be oxidized to quinone and react with amino groups and can promote antibacterial activity in alkaline environments. THY is a natural lipophilic monoterpene phenol, which can accumulate in the lipids of cell membranes and react with membrane proteins to destroy the structure and function of cell membranes and has excellent antibacterial activity under acidic conditions. The presence of OTA and THY enables TO/ASP hydrogel to maintain consistently high antibacterial activity over a wide pH range (4–9). The antibacterial rates of *E. coli* and *S. aureus* were 99.92% and 99.993%, respectively, in the pH range of 4–9. At the same time, hydrogels can effectively eliminate biofilm and destroy its structural integrity, and the biofilm biomass of *E. coli* and *S. aureus* decreased by 84.97% and 91.01%, respectively, compared with the PBS-treated group. The TO/ASP hydrogel prepared in this study is no longer affected by the change in wound pH and can realize high antibacterial efficacy in the whole process of wound healing. The functional richness of hydrogel was improved.

In the treatment of wounds, drug release decreases, and microorganisms may repopulate over time, so continuous controlled drug release is necessary. Ye et al. [63] designed a NIR-responsive photothermal hydrogel platform (ALG-HPR hydrogel) to control the drug-sustained release and achieve persistent antibacterial effects. First, the antibiotic rifampicin, photothermic agent indocyanine green (ICG), and fatty acids were encapsulated in natural halloysite clay nanotubes (HNTs). When irradiated with NIR, ICG converted light into heat, and phase transition occurred when the temperature rose above the melting point of fatty acids, followed by the release of rifampicin from HNTs, achieving drug release with NIR response. At the same time, the temperature does not exceed 42 °C, which will not burn the surrounding healthy skin tissue. Thus, through the synergistic effect with rifampicin, HNTs can achieve an antibacterial effect at a mild temperature. At room temperature, HNTs with a 25% drug-loading rate were cross-linked with alginate by Ca^2+,^ and hydrogels were obtained by in situ gelation. In vitro antibacterial experiment, rifampicin was continuously released under the control of photo-response of hydrogel after three-time of NIR irradiation, and the antibacterial efficiency against *S. aureus* was nearly 100%. After 21 days of treatment, the granulation tissue became thick, the epidermis regenerated, and finally, the wounds of the mice were almost completely healed. In short, ALG-HPR hydrogel can realize NIR-controlled release for long-term antibacterial performance and has great medical potential. This study proposes a new method of drug-controlled release, which provides insight into the continuous treatment of chronic wounds.

## 3. Summary and Prospect

In recent years, the research on natural-polymer-based hydrogels has made great progress. Natural organic polymers come from a wide range of sources whose composition and structure are similar to the extracellular matrix, and they have high biocompatibility and are easy to modify. Hydrogels can achieve a good antibacterial effect and eliminate microbial infection by loading antibacterial agents or combining them with other therapies, such as phototherapy. At the same time, through various modification methods, the hydrogel can have the ability to be anti-inflammatory and antioxidant and promote tissue regeneration and rapid wound healing. Each of the seven natural polymer materials introduced in this review has its own advantages and disadvantages. All of them can be prepared into ideal antibacterial hydrogels with appropriate modifications. However, the positive charge of CS can cause harm to microorganisms through electrostatic interactions. PDA can not only achieve photothermal sterilization but also obtain highly viscous hydrogels by bionic mussels. The inherent antibacterial properties of CS and PDA make them possible to be synergetically used with antibacterial drugs to improve the antibacterial ability of hydrogels. At the same time, these two materials can reduce the dosage of antibacterial drugs and improve biological safety. Therefore, CS and PDA have a broader application prospect in the preparation of antibacterial hydrogels. In short, natural-polymer-based hydrogels have excellent properties in all aspects and are still one of the research hotspots at present.

Although natural-polymer-based hydrogels have made good progress, they still have certain limitations. In the future, the development of natural-polymer-based hydrogels should be closer to the following trends: First, in the treatment process, the wound situation is constantly changing, so the function of hydrogels should also achieve intelligent regulation. For example, effective hemostasis should be provided during the hemostatic phase of the wound, and tissue regeneration should be induced through the release of growth factors or other methods during the proliferation phase. Secondly, hydrogels should have real-time monitoring and continuous release in response to antibacterial drugs because microorganisms have a strong proliferation ability, and secondary infections may occur during the process of wound healing. Third, the precise targeting of drugs is also extremely important because drugs have certain toxicity, which may affect healthy tissues and hinder the repair of wounds. Fourth, the functions of hydrogels should be diversified, such as antibacterial, antioxidant, anti-inflammatory, hemostatic, etc., to meet the needs of the whole process of wound healing. Finally, hydrogels should have excellent biocompatibility and biodegradability so that they can achieve clinical application. In summary, we hope that this review will give readers a systematic and detailed understanding of the current situation of the application of natural-polymer-based hydrogels in the treatment of infectious wounds in the past three years. At the same time, it is expected that this work can provide some guidance for the research of antibacterial hydrogels.

## Figures and Tables

**Table 1:** Summary of natural-polymer-based hydrogels for antimicrobial therapy.

Matrix Material	Superiority	Hydrogel	Modification	Highlight	Antimicrobial Activity	Mechanism	Reference
Chitosan (CS)	electropositive, effective killing of microorganisms by electrostatic interaction	BP/CS-bFGF hydrogel	carboxymethyl chitosan	Basic fibroblast growth factor was added to promote tissue regeneration.	*S. aureus*, *E. coli >* 67%	electrostatic interaction between carboxymethyl chitosan and bacteria	[50]
		CSG-PEG/DMA/Zn hydrogel	amidation reaction of polyethylene glycol monomethyl ether	be prepared by photoinitiated polymerization; multifunctional platform for antibacterial and anti-oxygen adhesion and hemostasis	*S. aureus, E. coli,* MRSA ≈100%	sustained release of the antimicrobial agent Zn^2+^	[51]
Polydopamine (PDA)	high viscosity; intrinsic photothermal properties	CAC/PDA/Cu(H_2_O_2_) hydrogel	CuSO_4_ and H_2_O_2_ accelerate the deposition rate of PDA	bionic mussels	*S. aureus* =99.87%; *E. coli* = 99.14%; MRSA = 99.25%	electrostatic interaction between carboxymethyl chitosan and bacteria; Sustained release of antimicrobial Cu^2+^	[52]
		CG/PDA@Ag hydrogel	in situ grown Ag; evenly disperse in guar glue hydrogel	Combined with photothermal therapy, the photothermal conversion efficiency of PDA is improved.	*S. aureus* =99.8%; *E. coli* = 99.9%	electrostatic interaction between guar gum and bacteria; sustained release of antimicrobial agent Ag^+^; photothermal effect of Ag@PDA	[53]
Gelatin	beneficial for fibroblast adhesion and growth	Gelatin-based hydrogel loaded with AgPOM	-	responsive to the acidic infectious environment	MRSA *>* 90%	photothermal effect of AgPOM; ^1^O_2_ formed by the reaction with H_2_O_2_	[54]
		GelMA-EGF/Gelatin-MPDA-LZM hydrogel	be amidated by methylacrylamide	photothermal and lysozyme synergistically remove biofilm and antibacterial activity	*E. coli* = 98.08%	photothermal effect of MPDA; lysozyme	[55]
Agarose	Stable thermally reversible hydrogels can be formed by physical cross-linking without the addition of cross-linking agents.	Gel1(Cyan)/Gel2(PCN) hydrogel	-	real-time monitoring; self-oxygenation enhances the photodynamic effect	*S. aureus, E. coli,* MRSA *>* 80%	photodynamic effects of oxygen-enhanced PCN-224	[56]
		CMA-Ag hydrogel	be amidated by carboxymethyl group	response to temperature and pH; The onset time of the inflammatory phase was earlier and the duration was shorter.	*S. aureus*, *E. coli* ≈ 100%	Sustained release of the antimicrobial agent Ag^+^	[57]
Hyaluronic acid (HA)	promoting granulation tissue regeneration and re-epithelialization; reducing inflammatory cell infiltration	HA-PEGSB-CMP hydrogel	be amidated by adipyl dihydrazide	good mechanical properties, can be used for motion wounds	EC, MRSA ≈ 100%	photothermal properties of the cuttlefish melanin nanoparticles	[58]
		BSP-U/DAHA hydrogel	be amidated by aldehyde group	Sol-gel transition can be achieved in response to photothermal and pH.	*S. aureus* =91.68%; *E. coli* = 94.94%	photothermal properties of hydrogels formed by cross-linking of catechol and Fe^3+^ ligands	[59]
Cellulose	Modifications can be made without compromising the structural and mechanical properties.	RPC/PB hydrogel	-	pH response intelligently releases the drug	*S. aureus* =84.3%	antimicrobial properties of resveratrol	[60]
		BC/GG-Cu@ZIF/GOx hydrogel	-	response to glucose	*S. aureus*, *E. coli* ≈ 100%	nanozymes consume glucose and generate ·OH antimicrobial agents	[61]
Alginate	nonimmunogenic and nonthrombotic	TO/ASP hydrogel	be amidated by diacetone acrylamide	not affected by environmental pH and has good antibacterial activity in the range of pH 4–9	*S. aureus* =99.92%; *E. coli* = 99.993%	antimicrobial properties of antimicrobial peptides, thymol, and oligomeric tannic acids	[62]
		ALG-HPR hydrogel	-	NIR responsiveness; long-term release of the drug	*S. aureus* ≈ 100%	photothermal properties of indocyanine green; antimicrobial properties of rifampicin.	[63]

## Data Availability

Data sharing not applicable.
